# Reintroducing the Effortless Assessment Research System (EARS)

**DOI:** 10.2196/38920

**Published:** 2023-04-26

**Authors:** Monika N Lind, Lauren E Kahn, Ryann Crowley, Wyatt Reed, Geordie Wicks, Nicholas B Allen

**Affiliations:** 1 Center for Digital Mental Health University of Oregon Eugene, OR United States

**Keywords:** mobile sensing, passive sensing, personal sensing, digital phenotyping, ecological momentary assessment, digital mental health

## Abstract

This paper reintroduces the Effortless Assessment Research System (EARS), 4 years and 10,000 participants after its initial launch. EARS is a mobile sensing tool that affords researchers the opportunity to collect naturalistic, behavioral data via participants’ naturalistic smartphone use. The first section of the paper highlights improvements made to EARS via a tour of EARS’s capabilities—the most important of which is the expansion of EARS to the iOS operating system. Other improvements include better keyboard integration for the collection of typed text; full control of survey design and administration for research teams; and the addition of a researcher-facing EARS dashboard, which facilitates survey design, the enrollment of participants, and the tracking of participants. The second section of the paper goes behind the scenes to describe 3 challenges faced by the EARS developers—remote participant enrollment and tracking, keeping EARS running in the background, and continuous attention and effort toward data protection—and how those challenges shaped the design of the app.

## Introduction

In 2018, we introduced the Effortless Assessment of Risk States (EARS) tool, a mobile sensing software that collects behavioral and interpersonal data via naturalistic smartphone use [1]. The original motivation for the development of the tool was to provide a new scalable approach for continuous measurement of behavior to facilitate the prediction of, and timely response to, mental health crises such as increases in suicidal thoughts and behaviors [1,2]. We designed the EARS tool to capture multiple indices of behavior by continuously collecting data from the various sensors in an individual’s personal smartphone during their normal use the device. This makes the solution highly scalable because there is no need to provide the user with special wearables or to ask them to change their current behavior by integrating a new device into their lifestyle. This substantially reduces the compliance burden associated with the measurement. We selected the indices measured based on findings that demonstrate their links to mental health states such as depression and suicidality [1]. These indices included physical activity, geolocation, sleep, phone use and duration, music choice, facial expressions, acoustic vocal quality, and natural language use [1]. Although the continuous collection of behavioral data from smartphones has been extensively used in some commercial applications, the EARS tool was specifically designed to only be used in research studies that are regulated by properly constituted Institutional Review Boards or Human Research Ethics Committees that require, among other things, fully informed consent.

**Figure 1:**
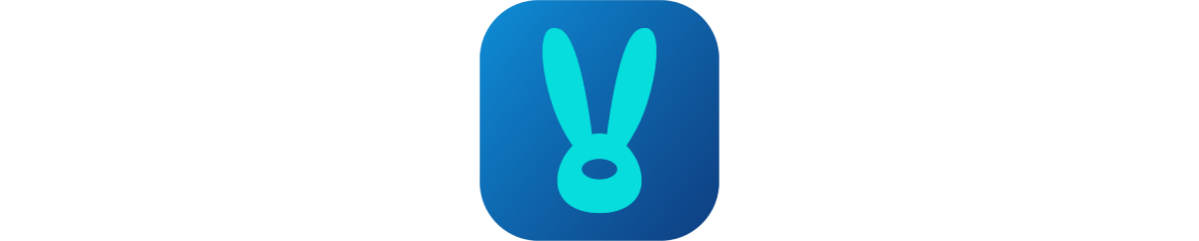
The Effortless Assessment Research System (EARS) app icon.

Much has changed since the launch of EARS, including its name. The acronym and the “bunny ears” icon remain (see Figure 1), but EARS now stands for the Effortless Assessment *Research System*. The name change not only reflects its broader application to a wide variety of use cases in behavioral research, in addition to our initial focus on the prediction of psychiatric risk, but also represents the extensive improvements made to EARS over the last 4 years. The first section of this paper will highlight those improvements via a tour of EARS’s capabilities across iOS and Android versions. The second section of this paper will go behind the scenes to describe 3 challenges faced by the EARS developers and how those challenges shaped the design of the app.

## What EARS Does

### Overview

The single most important improvement to EARS is the addition of a comparable iOS version to match the Android version. EARS on iOS is now programmed in Swift and is available on the Apple App Store (current version 2.1.2 at the time of this paper; search “EARS Mobile Sensing” in the Apple App Store). The iOS and Android versions share a set of core data streams (see Table 1). With minimal burden on the research participant, EARS can collect—with fully informed consent—data from a smartphone’s accelerometer, GPS, activity and motion (ie, walking, running, cycling, and driving), music and media listening, selfies, and text typed into the keyboard. For research teams who wish to collect the timing of typed text but not the content, both versions can collect the timing of keystrokes without collecting content. In addition, EARS allows researchers to administer custom ecological momentary assessment (EMA) surveys. In the Android version only, EARS can also collect app usage data and ambient light, although recent developments in iOS version 16 have opened up the possibility of collecting app usage in iOS using the Screentime application programming interface. This capability is current being built into the iOS version of EARS. In the iOS version only, EARS can also collect battery level, charge status, and call status. Researchers can decide which data streams to collect for their study, and we protect the confidentiality of these data with industry-standard encryption.

**Table 1:** Data streams collected by Effortless Assessment Research System (EARS) on Android and iOS and example behavioral features to which they can contribute.

Data stream	Android	iOS	Relevant behavioral features
Accelerometer	✓	✓	Physical activity and sleep
GPS	✓	✓	Patterns of mobility and sematic location
Activity and motion	✓	✓	Physical activity
Music and media	✓	✓	Musical and emotional qualities of music listened to
Selfies	✓	✓	Facial expression
Typed text: content and timing	✓	✓	Language content analysis (eg, sentiment) and keyboard kinematics
Typed text: timing only	✓	✓	Keyboard kinematics
EMA^a^	✓	✓	Self-report of experiences, activities, and contexts
App usage	✓	Coming soon	Extent and timing of phone usage, broken down into specific app categories
Ambient light	✓		Sleep patterns
Battery level		✓	Regularity of daily behavioral patterns
Charge status		✓	Regularity of daily behavioral patterns
Call status		✓	Frequency of social contacts

^a^EMA: ecological momentary assessment.

### Keystroke Data

The collection of text typed into the smartphone keyboard sets EARS apart from many other mobile sensing tools. All typed text, with the exception of anything typed into a secure field such as passwords and credit card numbers, are captured and stamped with the date, time, and application in which the keyboard is active. In other words, if a research participant sends a text message to their spouse while they have EARS installed, researchers can, with appropriate informed consent, know what they typed, when they typed it, and what application they used. (Obviously, these data are very privacy sensitive.) However, to protect the confidentiality of the recipient of the message (who has presumably not consented to having their data collected), there is no record of who received the message or their reply. On the Android version, EARS captures these data regardless of which keyboard a research participant uses. On the iOS version, EARS’s text capture depends on the participant using a custom EARS keyboard. The EARS keyboard for iOS has undergone a recent overhaul focused on improving its accuracy, autocorrection, and predictive text functions, as well as adding convenient features such as haptic feedback and trackpad mode (ie, long pressing the space bar for larger scrolling).

### EMA Surveys

Another recent, major update to EARS transferred control of EMA administration to the researcher. Previously, EARS staff had to program each survey via customized development that required many clarifications and iterations. Now, researchers use a convenient EARS dashboard to assemble their survey(s) from a set of item types (slider, true or false, single choice, multiple choice, the time of day, text entry, and informational text), define survey question content and logic (eg, branching), and customize the EMA schedule. EMA delivery schedules can be predetermined by researchers, but currently, event-based triggering on EMA surveys (eg, by specific patterns of mobile sensing data) is not available. Researchers also choose whether to enable several EMA-related functions, including EMA streak-based gamification (to increase compliance), scheduling bursts, and a risk alert function. The risk alert function, which automatically scores a completed survey and detects instances of predefined response patterns indicating high risk (eg, endorsing suicidal intent), is suitable for research with high-risk populations. When a predefined high-risk response pattern is detected, the system alerts study staff or clinicians by automatically sending a text message or email containing the information required to conduct a safety check with the participant.

### The Researcher Dashboard

The researcher dashboard itself represents a major update to EARS and serves 3 key functions. First, the dashboard facilitates the enrollment of participants and the installation of EARS on participant devices. Second, the dashboard enables data quality monitoring with daily reports on the specific data streams uploading from each participant. Third, as mentioned above, the dashboard allows researchers to design and administer EMAs.

### Deprecated Features

The development and maintenance of EARS requires flexibility to respond to changing operating system parameters and researcher needs. As such, updates to EARS have also resulted in the deprecation of several features. EARS no longer measures SMS text messaging frequency, nor does it capture in-call acoustic voice properties. These depreciations are in response to technical challenges and legal complexities. For example, the passive collection of voice data proved to be complex not only at the technical level (eg, it was difficult to reliably trigger the collection of voice data when the research participants were speaking into their phone) but also legally (eg, ensuring that the collection of the participant’s voice did not collect any voice data from a nonconsenting third party during phone calls). Because of our interest in emphasizing passive methods of assessment wherever possible, the video diary function (which required an active response from the participant) has also been phased out in favor of the selfie data stream. Sustaining EARS has also changed our approach to licensing and sharing open-source code. The requirements to maintain the software and support the implementation and maintenance of studies are very time-consuming. Although there are a number of free-to-the-user mobile sensing tools available, we have found that behavioral researchers are often looking for a solution that supports the implementation, administration, and analysis of these studies. As such, EARS is now licensed to a spin-out company, Ksana Health Inc, that can provide researchers with end-to-end support to conduct mobile sensing studies. This end-to-end support exceeded the capabilities of our university-based research center.

### Data Delivery to Researchers

At the conclusion of their study, researchers have the option to take delivery of EARS data in a raw form or as extracted features. In this context, “features” refer to metrics derived from the raw EARS data produced by participants. EARS data features tend to be more tractable for many research teams than raw EARS data. For example, our data scientists can take raw accelerometer data—of which a typical participant produces 4,000,000 records per day—and derive interpretable sleep features, including bedtime, waketime, and sleep duration. In addition, our data scientists derive location and travel features from raw GPS data, including but not limited to time spent at home, time spent traveling, and the number of travel events per day. Following the sentiment analysis approach of Byrne and colleagues [3], which showed a relationship between EARS-typed text and stress, our data scientists offer typed text features that measure positive sentiment and negative sentiment words. We also offer features that measure first-person pronoun use and absolutist language, which are related to mental health, especially indices of depression and suicide risk [4,5].

To date, over 10,000 people across Europe, the United Kingdom, North America, and Australia have participated in research studies using EARS. Study lengths vary from 1 week to 1 year of mobile sensing data collection. EARS has gathered mobile sensing data from participants on 88% of study days. Due to challenges discussed below, the iOS version lags behind the Android version with respect to data completion, with EARS on iOS collecting at least some mobile sensing data on 85% of study days versus EARS on Android’s 98%. Although EARS performs well on both platforms, the relative strength of EARS on Android supports its suitability for research conducted in low- and middle-income countries where Android dominates the mobile phone market.

The updates to EARS address a series of core challenges faced by EARS developers and researchers conducting studies using mobile sensing.

## Challenge #1: Participant Tracking

In all, 3 major challenges shaped the development of EARS. The first challenge, familiar to every researcher who has collected longitudinal data, was participant tracking. Early problems with participant tracking included unpredictable participant behaviors such as deleting or losing the EARS app or purchasing a new phone. In the pilot version of EARS, we had no way of detecting these behaviors in real time, which meant that we often did not discover missing data until the end of data collection. Remote installation of EARS was also impossible, so even in the case of a conscientious participant who notified us of their new phone purchase, the reinstallation of EARS required a laboratory visit or an email exchange with EARS staff. (EARS can easily be enabled on a new phone by reinstalling the app, but research staff need to be able to track the installation codes used for the installations on the old and new phones so that the participants data can be concatenated into 1 file for the final data analyses). In addition, to avoid causing headaches for research coordinators, participant tracking required EARS to ensure that the correct version of EARS is installed by participants, link mobile sensing data with established participant identifiers, and prevent unknown people from installing EARS and uploading mystery data. EARS developers have addressed these requirements by building a researcher-facing dashboard.

Among other capabilities, the dashboard allows researchers to set up their study, enroll participants, and monitor mobile sensing data uploads. Study setup includes choosing which sensors to collect and customizing the content and schedule of EMA questions, which is then built into the bespoke version of EARS for that study. The enrollment of participants entails the creation on the dashboard of a custom, single-use installation code, in both 16-digit, hexadecimal form and QR code form. This installation code ensures that the custom version of EARS—tailored to the choices made by researchers during setup—is installed only on the devices of consenting participants. The dashboard also interfaces with Amazon Web Services (AWS) and Qualtrics to enable EARS installation as part of remote informed consent.

**Figure 2:**
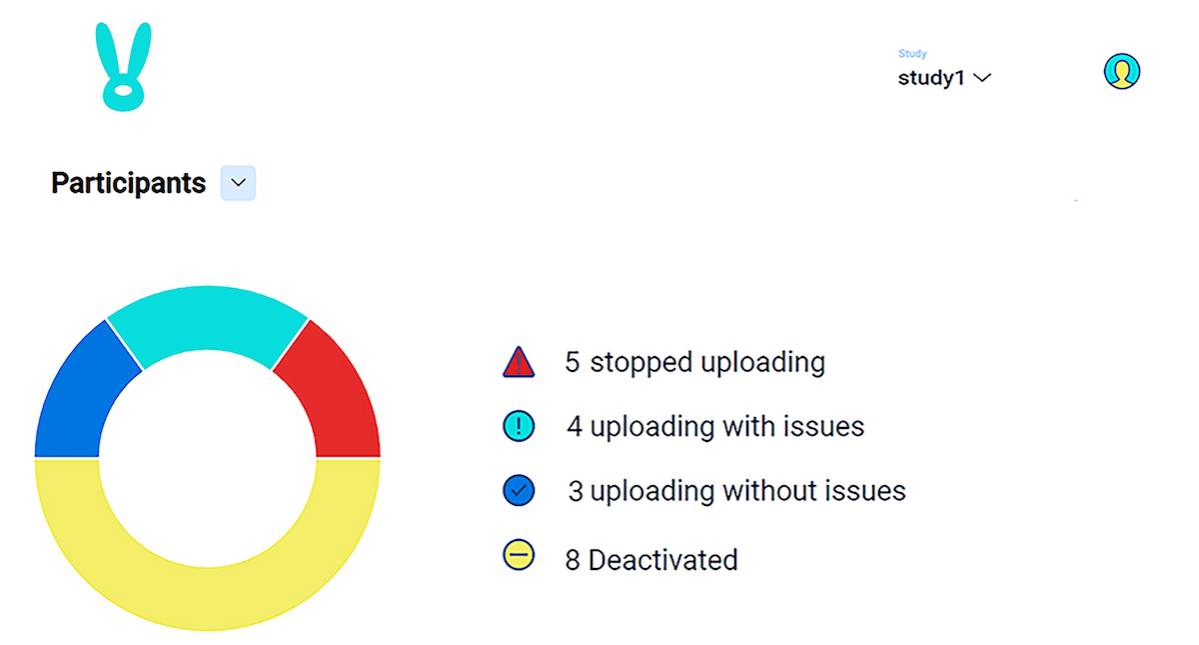
The overview conveys the number of participants uploading without issues, uploading with issues, not uploading at all, and deactivated. “Deactivated” denotes Effortless Assessment Research System (EARS) installations that have been deactivated by the researcher, usually when the participant finishes the study.

**Figure 3:**
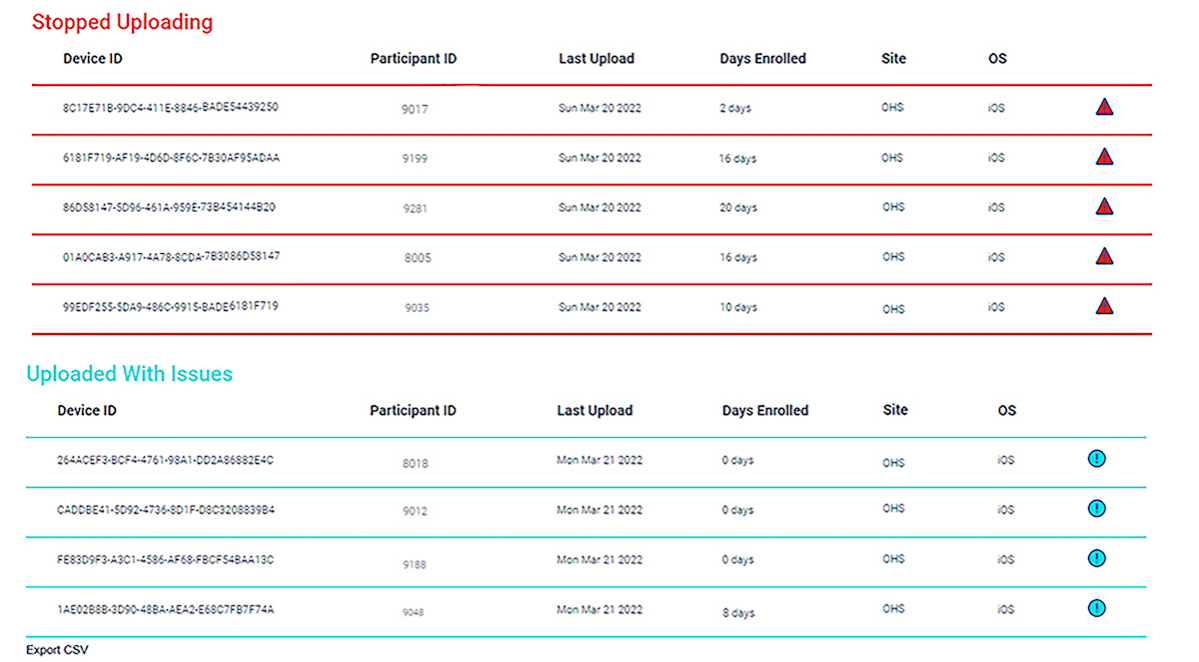
The device list elaborates on the participants categorized in the overview as uploading with issues and not uploading at all. The device list displays their participant ID, last upload date, research site, and operating system.

**Figure 4:**
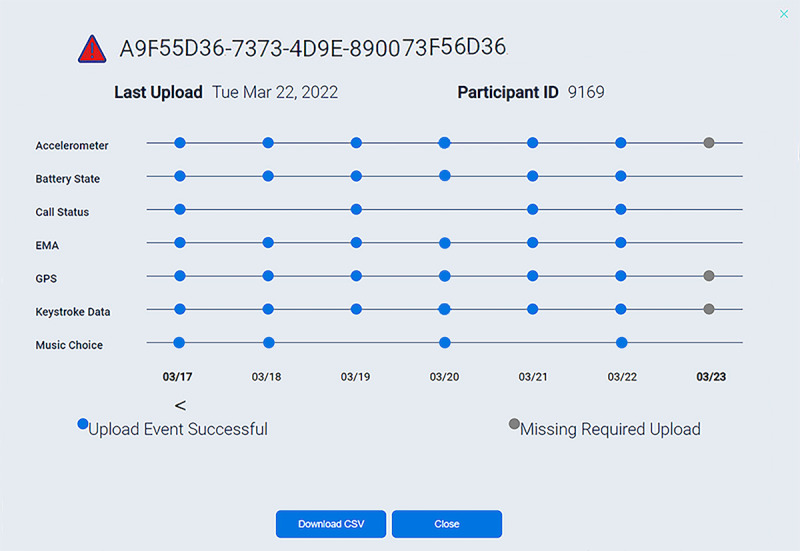
The device dot plot allows researchers to determine at a glance which sensors are uploading on which days for individual participants. EMA: ecological momentary assessment.

The monitoring of mobile sensing data uploads is facilitated by 3 figures on the dashboard: an overview (see Figure 2), a device list (see Figure 3), and a device dot plot (see Figure 4). The overview conveys the number of participants uploading without issues, uploading with issues (ie, missing expected sensors), and not uploading at all. The device list categorizes participant devices by status (eg, uploading without issues) and displays their last upload date, research site (important for multisite studies), and operating system. Finally, the device dot plot allows researchers to determine at a glance which sensors are uploading on which days for which participants. This information is crucial because many uploading issues are caused by participant behavior, which can be remedied by researcher reminders. For example, in iOS, it is possible for EARS participants to switch away from the EARS keyboard. If a participant does so, the dashboard will flag their device as not uploading text, which will signal the researcher to intervene.

## Challenge #2: Keeping EARS in Memory

The second challenge that shaped the development of EARS was keeping EARS running on participants’ smartphones during data collection. Battery optimization and privacy, among other concerns, have driven both the Android and iOS operating systems to close mobile apps automatically under certain conditions. These conditions include apps that run in the background without active user interaction and apps that draw on sensors such as GPS above a certain threshold, both of which are related to mobile sensing functionality. The severity of this issue on Android depends on the Android manufacturer (see the “Don’t kill my app!” website for more information [6]). A few Android handset manufacturers (eg, OnePlus and Huawei) account for the majority of the problems with keeping EARS in memory on Android. So far, the limited popularity of those manufacturers among our participants has contributed to the excellent performance of EARS on Android. iOS takes a more restrictive approach than Android.

After a relatively smooth Android-only launch of EARS in 2018, developing for iOS forced our programmers to solve 2 main problems. First, early testing on iOS revealed spotty collection of GPS and accelerometer data with evidence of the app getting repeatedly force-closed. It appeared that iOS took a dim view of our regular sampling of the GPS and accelerometer sensors. We adjusted by moving the GPS sensor to a threshold-based approach, that is, only collecting GPS data when the phone moved over 100 meters. We now apply this distance-based threshold in both iOS and Android versions. For the accelerometer, we changed collection to a retroactive application programming interface rather than collecting in real time. Not only did these changes prevent EARS from getting automatically closed by iOS, but they also reduced the impact of EARS on battery life.

The second main problem was that iOS optimizes user experience by automatically closing unused apps over time. Although the time varies among device models and user usage habits, on average, this results in most apps being closed after approximately 24 hours without active use. This means that EARS research participants must interact with the EARS app once per day to ensure ongoing sensor data collection. We addressed this problem by requiring that all research studies send 1 prompt to open the app to participants per day. The least onerous but still adequate version of this prompt requires just 1 tap: participants simply acknowledge a notification from EARS, which brings EARS to the foreground, although most studies take the opportunity to collect a short EMA survey at this time. An upside of this requirement is that it maintains participants’ awareness of EARS running on their phones, thereby strengthening ongoing informed consent.

Common to Android and iOS, a third aspect of the challenge of keeping EARS in memory was training researchers to train participants. Continuous data collection by EARS depends on participant behavior, and researchers are the primary point of contact for participants. As such, it is critical that researchers receive comprehensive training from the EARS team. For example, in a scenario in which an iOS user switches away from the EARS keyboard, the researcher detects the switch from the dashboard and can prompt the participant to switch back to the EARS keyboard to ensure ongoing data collection. Ultimately, the goal is to achieve as close to continuous mobile sensing data collection as possible, and the participant’s ability to facilitate this goal depends on the researcher’s fluency with the dashboard and the participant’s fluency with enabling EARS capabilities—both of which are supported by the extensive training, resources, and support provided by the EARS team.

## Challenge #3: Data Protection

A third challenge that continues to shape the development of EARS is the protection of participant data. Many EARS researchers study adolescents, which increases the importance of rising to this challenge. Our recent data protection efforts have focused on 2 issues: encryption protocols and compliance with the with best practices and statutory requirements.

Our encryption protocols changed after careful consideration of likely threats to data protection. Per advice from data security professionals, we decided that the storage of the data in multiple locations presented a significant threat to data security. To address this increased risk, we made changes that minimized the storage locations and transfers. In short, we reduced our encryption layers from 2 to 1 and took advantage of the cloud-based data processing capabilities afforded by our cloud service, AWS. In our current approach, the data are single-encrypted in transit from the phone to the cloud. Upon arrival on the cloud, the data are decrypted then re-encrypted with AWS’s standard server-side encryption. Data processing and feature extraction occur on the cloud without ever having to manually decrypt data, instead taking advantage of AWS’s built-in decryption (and subsequent re-encryption) when temporarily bringing data into memory for processing. Researchers take delivery of their raw data and features in a single-encrypted transfer from the cloud. Some researchers opt to receive only extracted features to avoid ever having raw mobile sensing data stored on their local systems. Our long-term goal remains to provide researchers with the option to constrain data processing and feature extraction to the phone itself, thereby making the phone the exclusive home of the raw data. In the meantime, the cloud servers where EARS data live are also managed by a cybersecurity analyst employed by Ksana Health.

Ksana Health has now completed a third-party assessment for security and privacy controls (System and Organization Controls [SOC] 2 Type 2). A SOC 2 Type 2 report is an internal controls report capturing how a company safeguards data and how well those controls are operating. Companies that use cloud service providers use SOC 2 reports to assess and address the risks associated with third-party technology services. EARS received unqualified approval attesting to the strength of data security controls and compliance with best practices and statutory requirements, including the following:

- American Institute of Certified Public Accountants (AICPA), Trust Services Criteria
- Healthcare Insurance Portability and Accountability Act (HIPAA) Security Rule
- HIPAA Privacy Rule
- General Data Protection Regulation (GDPR) Controls Mapping

To highlight the details of one of these requirements, the European Union’s adoption of the GDPR in 2016 and its enforcement starting in 2018 required us to ensure that EARS meets those standards. To the extent that the information we collect is health data or another special category of personal data subject to GDPR, we ask users for their explicit consent to process the data. We obtain this consent separately when they enroll in a study. Additionally, users can use the account settings to withdraw consent at any time, including by stopping the use of a feature, removing our access to a third-party service, unpairing the device, or deleting the data or the account. Users can also uninstall the app at any time, which halts data collection. We support advancements in data and privacy protections, and we expect regulations around the world to follow the European Union’s lead. In fact, California already has with the California Consumer Privacy Act. As such, we view evolving data protection regulations as a challenge that will continue to improve EARS and mobile sensing research in general.

## Future Directions

We hope that the increasing the adoption of EARS by mobile sensing researchers will enable us to develop EARS not just in response to challenges but in the pursuit of innovation. Mobile sensing opens up the possibility of collecting behavioral data in a highly scalable way that is continuous, ecological, and objective without creating significant participant burden. These data can be used to provide answers to questions that were not previously addressable with self-report or laboratory-based methods. However, to achieve this goal, we must have tools that address researchers’ needs while respecting participants’ rights to privacy and data security. EARS represents one attempt to continuously improve these tools to achieve these goals.

## Abbreviations

AICPA: American Institute of Certified Public Accountants
AWS: Amazon Web Services
EARS: Effortless Assessment Research System (formerly Effortless Assessment of Risk States)
EMA: ecological momentary assessment
GDPR: General Data Protection Regulation
HIPAA: Healthcare Insurance Portability and Accountability Act
SOC: System and Organization Controls
